# Revisiting the seven pillars of RDoC

**DOI:** 10.1186/s12916-022-02414-0

**Published:** 2022-06-30

**Authors:** Sarah E. Morris, Charles A. Sanislow, Jenni Pacheco, Uma Vaidyanathan, Joshua A. Gordon, Bruce N. Cuthbert

**Affiliations:** 1 National Institute of Mental Health, Neuroscience Center, 6001 Executive Blvd, Bethesda, MD 20892 USA; 2 Wesleyan University, Middletown, USA; 3 Present affiliation: Boehringer Ingelheim, Ingelheim am Rhein, Germany

**Keywords:** Psychiatry, Diagnosis, RDoC, Research Domain Criteria, NIMH

## Abstract

**Background:**

In 2013, a few years after the launch of the National Institute of Mental Health’s Research Domain Criteria (RDoC) initiative, Cuthbert and Insel published a paper titled “Toward the future of psychiatric diagnosis: the seven pillars of RDoC*.*” The RDoC project is a translational research effort to encourage new ways of studying psychopathology through a focus on disruptions in normal functions (such as reward learning or attention) that are defined jointly by observable behavior and neurobiological measures. The paper outlined the principles of the RDoC research framework, including emphases on research that acquires data from multiple measurement classes to foster integrative analyses, adopts dimensional approaches, and employs novel methods for ascertaining participants and identifying valid subgroups.

**Discussion:**

To mark the first decade of the RDoC initiative, we revisit the seven pillars and highlight new research findings and updates to the framework that are related to each. This reappraisal emphasizes the flexible nature of the RDoC framework and its application in diverse areas of research, new findings related to the importance of developmental trajectories within and across neurobehavioral domains, and the value of computational approaches for clarifying complex multivariate relations among behavioral and neurobiological systems.

**Conclusion:**

The seven pillars of RDoC have provided a foundation that has helped to guide a surge of new studies that have examined neurobehavioral domains related to mental disorders, in the service of informing future psychiatric nosology. Building on this footing, future areas of emphasis for the RDoC project will include studying central-peripheral interactions, developing novel approaches to phenotyping for genomic studies, and identifying new targets for clinical trial research to facilitate progress in precision psychiatry.

## Background

More than a decade has passed since the National Institute of Mental Health (NIMH) Research Domain Criteria (RDoC) project was initiated, and RDoC has opened a door for researchers who strive to move beyond diagnostic syndromes derived by clinical description as the starting point or the outcome measure(s) for research on mental illness. RDoC has helped to expand the conversation about how psychopathology research is carried out and introduced new ways to conduct it. Some researchers have embraced the principles of RDoC, while others have challenged it in ways that have helped it to evolve. Psychopathology research has diversified in various ways over the past decade. New approaches include studying common dimensions across a combination of disorders pooled together (e.g., across the psychosis spectrum; [1]); researching a particular feature within a heterogenous disorder, such as blunted reward processing in melancholic depression [2] or brain connectivity in schizophrenia [3]; carrying out research with new dependent variables, for instance, changes in striatal activity in the study of anhedonia [4]; and researching relevant variables independent of existing diagnostic classifications [5].

The existence of several major initiatives to reframe psychiatric diagnosis, including the Hierarchical Taxonomy of Psychopathology model [6], the network approach [7], and the clinical staging model [8], illustrates the ongoing need for new approaches to better understand psychopathology. It has become clear that syndromal diagnoses as defined in the American Psychiatric Association’s Diagnostic and Statistical Manual (DSM; [9]) and the World Health Organization’s International Classification of Disease (ICD; [10]) for the past four decades, while emphasizing reliability, have not borne out the identification of valid mechanisms. A more complete understanding of the disruptions in the systems that interact between biology and behavior and their relation to palpable psychopathology is needed for the development of novel therapeutic agents [11] and important to sharpen psychological interventions as well [12]. Perhaps the highest hurdle faced in overcoming problems in psychiatric nosology concerns the extent that diagnoses have been reified — seen as “real entities” — when in reality they are not natural kinds [13]. The reification of clinical syndromes left clinicians and researchers alike with an epistemic roadblock. By providing reference points for third-party payers and disability adjudications, as well as for regulatory bodies, clinical diagnostic manuals face practical constraints on the degree to which they can modify their descriptions and criteria. We (and others) have argued that researchers need a framework that is independent of such constraints in order to facilitate progress connecting advances from integrative neuroscience with disordered behavior and to obtain the knowledge that can help improve clinical diagnostic manuals.

Cuthbert and Insel described the “seven pillars of RDoC” in this journal in 2013 [14] and those principles have proven to be the sustaining foundation of the framework. These principles have roots in experimental psychopathology [15] and align with longstanding aims to make empirical and conceptual connections across multiple measurement methods [16]. Detailed descriptions of the rationale and development of RDoC have been provided elsewhere [17, 18]. In brief, RDoC was developed to provide a framework for psychopathology research that was not yoked to the traditional diagnostic syndromes and to support a dialog for consensus among members of various constituencies (research grant applicants, members of peer review committees, funding and regulatory agencies, and journal editors) in the scientific community for scientifically sound research not bound to the status quo. The relationships between RDoC and diagnostic manuals and the role of RDoC in NIMH research funding have both become clearer since RDoC was launched. Specifically, existing diagnostic criteria remain the standard for clinical use, while research that informs clinical decision-making and may inform future changes to diagnostic practice and criteria (including research that adopts RDoC principles) carries on concurrently. NIMH never stopped funding research focused on existing diagnostic categories but encouraged investigators to critically examine their assumptions about diagnosis-based classification and to consider alternative approaches. Such work has been stimulated and supported in part via funding opportunities specifically designated for RDoC research, and RDoC-focused grant applications have also competed well in the general pool of applications.

As one marker of progress toward RDoC’s goals, over one thousand papers have resulted from grants funded under the seventeen RDoC-focused funding opportunities published by NIMH. It could be asked whether researchers are adopting the RDoC approach for pragmatic reasons, rather than scientific, if they perceive that doing so confers an advantage in the competition for NIMH research funding. We are reassured by the support of funding agencies outside of the USA for dimensional approaches that the uptake of RDoC (and RDoC-like) approaches is not entirely attributable to NIMH demand characteristics. The European Union’s Roadmap for Mental Health Research in Europe (ROAMER) project [19], the Innovative Medicine Initiative’s recently renewed Psychiatric Ratings using Intermediate Stratified Markers (PRISM) project [20, 21], and the Wellcome Trust’s Multi-Channel Psych initiative to stratify depression patients and match to treatment [22] are examples of international interest.

To mark the first decade of RDoC, we revisit here the seven pillars of RDoC in the context of the evolution in perspectives that has followed since these principles were conceived, highlight recent research projects that exemplify RDoC principles, summarize updates to the framework and other RDoC activities, and discuss areas for potential future emphasis.

## Main text

### Pillar 1: The translational perspective: psychopathology research should start with what is known about normative neurobehavioral processes

**Fig. 1:**
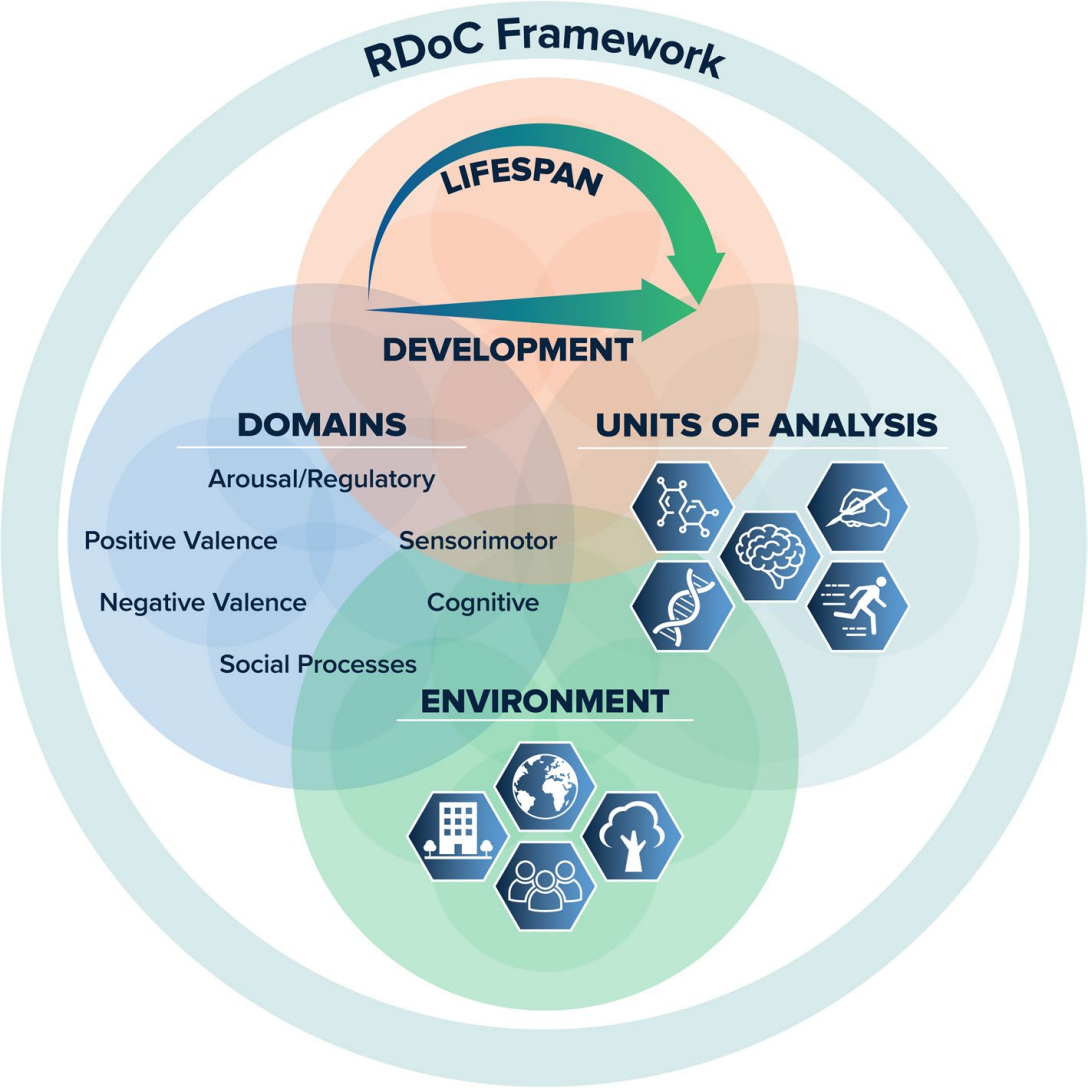
The RDoC framework provides an organizational structure for research that considers mental health and psychopathology in the context of major domains of basic human neurobehavioral functioning. The framework currently includes six major functional *domains* with associated constructs, which are studied along the full range of functioning from normal to abnormal. Both behavioral and biological aspects of functioning change and mature throughout childhood/adolescence and across the life span, and so research on *development* is essential. Equally important is the study of various aspects of the *environment*, including the physical environment, cultural components, and factors such as social determinants of health. The RDoC framework encourages researchers to measure and integrate many classes of variables (*units of analysis*, e.g., behavioral, physiological, and self-report data) in order to seek a comprehensive understanding of the construct(s) under study

The foundational pillar of RDoC is that the starting point for research on mental illness should be translational understanding drawn from basic science of the functions (such as attention or response to threat) that can be variously characterized by neural, behavioral, cognitive, and other systems, with disorders examined as disruptions in these functions resulting in dysfunction of varying degrees. The functions are termed “constructs,” consistent with longstanding usage [23], and are grouped into superordinate domains that contain multiple related constructs (such as cognitive systems). Constructs can be quantified using various levels of analysis (including neural circuit-based measures, behavior, and self-report). These levels (termed “units of analysis” in the framework, see below) are rooted in translational science and are intended to provide a link from basic to clinical research (Fig. 1).

One prominent example of this translational pipeline is in the area of predictive coding, that is, the theory that the brain continuously updates its models of the environment on the basis of new information. Informed by basic research focused on dopamine signaling and learning, this formal, quantitative model is well-elaborated, allowing highly nuanced simulations and modeling to test effects of variation in numerous perceptual and cognitive parameters. This model supports translational work focused on testing new hypotheses about the disruption of processes involved in predictive coding in the psychosis spectrum [24, 25], including in individuals who experience auditory hallucinations but do not meet diagnostic criteria for the schizophrenia syndrome [26] and those who experience trauma-related hallucinations [27]. As another example, work built upon basic behavioral and neuroscience research in humans, rodents, and non-human primates has contributed to a more nuanced understanding of anhedonia as a multi-component phenotype that spans psychiatric conditions [28, 29] and may contribute to heterogeneity within major depressive disorder [30].

Reciprocally, by focusing on systems and processes rather than nosological entities, RDoC boosts the translational potential of animal research for addressing neurobiological questions relevant to psychopathology rather than models of specific mental illnesses [31]. The RDoC framework “allows one to evade a major challenge of translational studies of strict disease-to-model correspondence” [32]. As an example, a battery of behavioral assays that map onto constructs in RDoC’s Positive Valence Systems and Negative Valence Systems domains “enable[es] the integrated study of motivational processing of rewarding and aversive stimuli in [mice]” [33]. Much of this type of work is suited to the application of biophysically realistic neural-network models [34]. It is important to bear in mind, however, the assumptions that are implicit in this emphasis on translational research, including the risk that by “focus[ing] on neural circuits seen throughout phylogeny, [RDoC] is likely to neglect quintessentially human phenomena that are remarkably important for understanding humans (including the development of psychopathology and its potential treatment)…One cannot study in rats the belief that one is worthless” [35]. RDoC does not attempt to restrict the focus of study to only those elements that have human-to-animal homology, and certain units of analysis (e.g., self-report vs behavior vs circuits) are more useful and appropriate for a given approach (such as animal research) than others.

### Pillar 2: The dimensional approach: assume dimensionality — among disorders and between illness and health — unless data show otherwise

Over the last few decades, the notion that mental disorders and neurobehavioral constructs associated with them are likely dimensional in nature has come to be increasingly accepted, as reflected in a comment in an editorial in a major schizophrenia journal noting that “...emerging change in research priorities reflects a new emphasis on porous diagnostic boundaries with increased attention to similarities and differences between disorders. Also, a focus on deconstructing heterogeneous clinical syndromes in order to identify specific elements of pathology is advancing science, often in a dimensional framework without diagnostic specificity” [36]. RDoC explicitly encouraged the study of biobehavioral constructs that “[D]etermine the full range of variation, from normal to abnormal, among the fundamental components to improve understanding of what is typical versus pathological” [37]. Once these dimensions — and the relationships among them — are more fully understood, it will be possible to empirically define cut-points, clusters, or subgroups that are informative for clinical decision-making and predictive, for example, of prognosis or treatment response.

Dimensional approaches have started to yield an improved understanding of neurobehavioral processes that are relevant for dissecting heterogeneity within a diagnostic category and identifying commonalities across disorders. For example, Lang and colleagues measured fMRI activity in the amygdala and ventral visual cortex during viewing of emotionally evocative scenes in a large transdiagnostic sample of patients with various mood and anxiety disorders (plus healthy controls). A dimension of emotional reactivity was observed in the patients, ranging from highly blunted to highly reactive as compared to controls; furthermore, reactivity was inversely related to a trauma factor score [38]. These results illustrate the complex dimensions that distinguish “typical versus pathological” and the importance of effects such as trauma that have strong effects in a transdiagnostic manner. Similarly, shared abnormalities have been found in brain structures across major depression, schizophrenia, bipolar disorder, and obsessive-compulsive disorder [39]; in neural connectivity in schizophrenia and bipolar disorder [40]; and in behavioral impairments in executive function across attention-deficit hyperactivity disorder and autism spectrum disorder [41]. These — and other — new transdiagnostic findings stand out against the background of a research literature that continues to consist largely of studies focused on single diagnostic categories with little consideration of diagnostic heterogeneity. It is challenging to reconcile transdiagnostic findings with the many reports of robust and reliable differences between individual patient groups and healthy individuals and differences among patient groups. Studies focused on a single disorder can give the impression of diagnostic specificity but until that is directly tested in transdiagnostic work, it is important to be cautious. One way to approach this challenge is to think about diagnostic labels as proxy variables. Just as sex can be an imperfect proxy for genetic, endocrine, anatomical, and other variables, it is important to ask whether a diagnosis of schizophrenia, for example, is serving as a proxy for psychosis or some other neurobehavioral abnormality for the purpose of answering a research question and whether a more precise measure might yield a more specific answer. For instance, schizophrenia is often viewed as a disorder of severe cognitive impairment. However, a recent study of multiple biotypes derived from data-driven analyses of psychotic spectrum disorders reported that 28% of patients with a schizophrenia diagnosis (and 39% patients with schizoaffective disorders, often lumped with schizophrenia in research studies) did not differ significantly from healthy controls on cognitive measures; in contrast, 46% of patients with psychotic bipolar disorder — typically regarded as an affective disorder – were severely or moderately impaired on cognitive measures [42]. Thus, a challenge for future dimensional research is to continue to refine the phenotypes utilized in such studies, rather than using diagnostic categories as anchors. In service of this, continued efforts to chart the landscape of associations and differentiations among symptoms, behaviors, and mechanisms, within and across diagnoses, are needed.

### Pillar 3: Reliable and valid measures are needed to dissect heterogeneity

The third pillar comes straight from the original NIMH Strategic Goal, to “develop reliable and valid measures of these fundamental components of mental disorders for use in basic studies and in more clinical settings” [37]. It was noted in the original paper that measurement development was a high priority for RDoC research and that well-validated and psychometrically optimized behavioral tasks with foundations in cognitive neuroscience were beginning to appear [43]. Since then, NIMH has made strides to assess the availability and utility of such tools and to encourage critical work needed to generate new measures. To hasten the development of standardized paradigms and measures, NIMH convened a workgroup to review the availability and utility of tasks that assess the constructs in the RDoC matrix and make recommendations about future work on measurement development. A specific challenge identified during this meeting was the shortage of normative data and information about psychometric properties (such as test-retest reliability) for many available tasks [44]. The workgroup made recommendations about tasks for assessment of most RDoC constructs; however, it was clear that additional investment in task development work was needed. In 2018, NIMH published a funding opportunity announcement encouraging grant applications focused on empirical optimization of existing tasks or development of new behavioral tasks to measure RDoC constructs [45]. NIMH priorities for task development include the use of computational approaches that allow testing and refinement of models when compared against actual data (e.g., [46]).

Over the past 10 years, digital technologies have emerged that fundamentally change the way we are able to collect data [47] and applications of these tools to modernize the assessment of cognition are underway. The National Institutes of Health (NIH) Mobile Toolbox, for example, allows remote self-administration of cognitive tests [48]. Combined with new techniques, including ecological momentary assessment and passive monitoring, scientists are now able to capture data in more natural settings and in near real time to supplement traditional assessment methods [49–51]. The ability to apply these techniques to data collection in large, population-based samples is a critical step toward dissecting the heterogeneity that is prevalent in psychiatric disorders.

### Pillar 4: Novel research designs and sampling methods are needed to elucidate data-driven phenotypes

The fourth pillar highlights the notion that accomplishing the goals of RDoC will necessitate new study designs and sampling strategies for mental health research. The common approach of using traditional clinical diagnoses to define a group and comparing the group to healthy control subjects on the measure(s) of interest tends to perpetuate assumptions about the homogeneity of these groups. Given RDoC’s focus on dimensions of functioning that cut across disorder boundaries and the priority to understand rather than ignore the inherent heterogeneity within mental health disorders, RDoC asks the field to reconsider how to set up rigorous research questions. For example, an ongoing project is using innovative methods to identify non-help-seeking individuals who are socially disconnected in order to examine contributors to social disconnection — such as social processing ability and social motivation — along the health-to-illness continuum, including in people experiencing psychosis [52, 53].

Recent work in the area of attention-deficit/hyperactivity disorder (ADHD) is useful to showcase how RDoC principles might be applied to study heterogeneity within disorders. The clinical presentation of ADHD is heterogenous, with extensive variability in phenotypes, suggesting that there might be meaningful subgroups and/or dimensions within ADHD [54–57]. Work has focused on classifying the heterogeneity within the ADHD population, trying to identify subtypes and profiles that are helpful to exemplify individuals at greater risk for persistent ADHD, those who may respond well to certain treatments, and those whose symptoms may remit during adolescence. An exploration of dimensional measures of executive functioning like working memory has uncovered multiple trajectory classes in both the ADHD and typically developing populations, as well as distinct relationships between cognitive processes and ADHD symptom change [58]. Specifically, the rate of naturalistic change in working memory predicted symptom remission in ADHD. Working memory impairment that failed to resolve before adolescence was correlated with persistence of ADHD symptoms through adulthood, whereas if working memory performance improved to age-appropriate levels, participants typically experienced a remittance of symptoms.

A similar exploration using computational methods to analyze emotion trait profiles revealed three subtypes within a large sample of children with ADHD: “mild” (normative emotional functioning), “surgent” (high positive affect), and “irritable” (high negative affect). Subjects in the irritable group showed the greatest symptomatic stability over time, exhibiting a more severe and persistent set of ADHD symptoms, and this subtyping was a better prospective predictor of clinical outcomes than standard baseline indicators [59]. When combined with cognitive profiles, the surgent and irritable groups were easily split into a group with and without cognitive deficits. Those children who displayed an irritable emotional profile and cognitive deficits had the most severe ADHD symptoms [60]. Further exploration of these subtypes and polygenic risk scores (PRS) for both ADHD and major depressive disorder (MDD) showed that despite a high association between PRS for ADHD and MDD, these emotional dysregulation profiles were more strongly associated with ADHD PRS than MDD PRS, suggesting that the subtypes represent distinct pathways via which genetic risk might lead to disorder [61]. This line of research demonstrates that using integrative, dimensional approaches has helped to identify subtypes of the disorder, which may be more informative to treatment and intervention practices.

### Pillar 5: RDoC encourages integrative methods rather than favoring one method over another

RDoC encourages studies that integrate multiple measurement classes (e.g., behavior, self-reports, neural systems, or genetics), with the particular measures chosen for any given study depending on the research question. Furthermore, the emphasis is upon relations among variables rather than prioritizing one kind of observation over another; for example, the term “units of analysis” was deliberately chosen over “levels of analysis” for referencing measurement classes so as to not inadvertently imply reductionism. A major goal in this regard is to address the mind-body constraints that have historically plagued clinical and research understandings of mental disorders, generating an oversimplistic reductionistic approach to disorders and discouraging more careful approach to brain-behavior relationships. The criterion that RDoC constructs should have evidence for both neural circuits and functional behaviors in order to address mind-body issues has somewhat ironically resulted in criticism that the RDoC framework is reductionist due to its inclusion of biological measures (see [62] for a discussion of this critique). In fact, philosophers of science accept with no qualms that accounting for biological variables does not diminish the value of behavioral observations (see [63]).

While traditional research designs are organized in terms of diagnostic groups or particular symptoms, one aspect of integrative approaches is to specify independent variables from other measurement classes. For instance, a recent transdiagnostic study of anxiety disorders grouped patients on the basis of their physiological reactions during an imagery assessment [64]. Patients imagined personal fear scenes and neutral scenes, during which a composite measure of heart rate and startle potentiation responses was determined for each trial; the difference between responses during personal fear scenes and neutral scenes was computed for the reactivity score, and all patients were ranked in order to obtain five quintiles of reactivity. Reactivity scores were inversely related to functional impairment in nearly linear fashion, rather than the positive relationship that might be expected if hyperreactivity reflected greater fear. Similar to the “proxy” findings noted above, the primary diagnosis bore only a modest relationship to the hyperreactive-to-hyporeactive dimension, with about 70% of the former diagnosed with circumscribed fear (e.g., specific phobia) and an approximately equal percentage of the latter with anxious-misery disorders (e.g., generalized anxiety disorders). Such a design is well-suited to explore phenomena that cut across multiple disorders.

It should be acknowledged that at this stage of the science, the integrative specification of the complex interrelations in psychopathology is no simple task. Pathways from genes to behavior, for example, are both divergent and convergent such that the relationships between observations in these systems will be probabilistic and pleiotropic [65, 66]. Parallel work directed toward overcoming such complexities of the integration goal includes methods that employ computational neuroscience to examine bi-directional links among different types of measures and identify regularities, irregularities, and other features [67]. Dynamical systems modeling is one of the many current areas of progress, which can model the relationships among many measurement classes as they vary across time in order to provide more elaborated accounts of how systems interact during both short-term processing and longer-term behavioral or symptom patterns [68].

### Pillar 6: RDoC’s scope is constrained to focus on constructs for which there is solid evidence to serve as a platform for ongoing research

As noted in the original “seven pillars” paper [14], RDoC is not intended to curate a comprehensive set of clinical features that encompass the many symptoms and signs for which individuals may seek treatment; rather, the aim is to encourage psychopathology research that frames hypotheses in terms of neurobehavioral constructs rather than groupings based on predetermined diagnostic criteria. In other words, RDoC is intended to generate a literature that can (among other goals) inform future versions of diagnostic systems rather than create an alternative clinical manual. RDoC is sometimes described as an alternative to existing diagnostic systems, but such framing erroneously implies a shared scope and purpose. RDoC is narrower in scope than diagnostic systems and serves a specific research purpose. Such research yields novel ways of stratifying, classifying, and clustering psychopathology, and the validity of these can be tested by examining their ability to predict prognosis or treatment response (thus crossing paths with the purpose of diagnosis); however, further work would be needed to develop diagnoses informed by these novel characterizations. RDoC domains and constructs, in and of themselves, do not necessarily define valid clinical entities for the purposes of clinician communication, drug development, or regulatory processes but the framework serves as a roadmap via which translational behavioral neuroscience research may converge with diagnostic practice [69].

Because of the enormous pool of constructs that have been the focus of study in psychology and psychiatry that could be brought to bear on the goals of RDoC, it was necessary to constrain the constructs considered for inclusion in the RDoC matrix to those which met the criteria described above. These criteria have not been systematically operationalized and can reasonably be critiqued as under-specified [70]; however, they provide guardrails against excess proliferation of constructs and serve as guiding principles to anchor RDoC’s focus on translational research.

### Pillar 7: RDoC is flexible and dynamic to accommodate the research advances that it tries to foster

The RDoC framework was proposed as a means to free researchers from the constraints of the diagnostic system, in order to design research studies that would push our understanding of these boundaries. Accordingly, the final pillar addresses the need for a research system such as RDoC to have flexibility in dynamically accommodating those research advances that it tries to foster. From the outset, the RDoC framework was depicted as a matrix intended to offer a starting point for scientists to understand the goals and scope of the RDoC initiative. The elements listed in the matrix, including the five domains (Negative Valence Systems, Positive Valence Systems, Cognitive Systems, Social Processes, and Arousal and Regulatory Systems), constructs associated with each domain, and cross-cutting units of analysis, were put forth as exemplars, with an assurance that the matrix would evolve over time as new research findings came to light. A workgroup focused on changes to the RDoC matrix was convened by the NIMH Advisory Council in 2016 [71] and this group has overseen two substantial changes. The first was a reorganization of the constructs within the Positive Valence Systems Domain [72], and the second was the addition of a sixth domain focused on Sensorimotor Systems [73].

In spite of a decade of changes, it is possible that RDoC has become over-reliant on the matrix. The pace of science has become so fast that it is extremely difficult to maintain the process of evaluating and curating new domains, constructs, and methodologies. Accordingly, the matrix risks ending up in the midst of another prescriptive system that is antithetical to its goal. Although thoughtful effort has been put into the changes, we have simultaneously begun to de-emphasize the specific content and structure of the RDoC matrix. Rather, we encourage investigators to consider the domains, constructs, and elements of the matrix to be exemplars and to focus on the principles of the framework (e.g., brain-behavior constructs, dimensional functions, and integrative analyses) within the context of environmental factors and developmental processes in considering their research plans. This shift in emphasis away from the matrix and toward a more holistic concept is reflected in the recently updated graphic depiction of the framework in Fig. 1.

## Summary and discussion

Reflecting at this milestone, it is clear that the seven pillars have provided a strong foundation for the first decade of the RDoC initiative and that RDoC has served an important role in a rigorous and productive scientific conversation about psychiatric diagnosis and diagnostic validity. Looking toward the future, we highlight here some possible future directions and areas of increased emphasis for the initiative.

RDoC encourages the study of mental disorders using integrative methods including cellular and molecular, circuit-based, behavioral, and self-report measures but has put less emphasis on measures and mechanisms from outside of behavioral neuroscience. New discoveries linking other biological systems to behavior and psychiatric symptoms present expanded opportunities in the mind-body space. For example, new understanding about how immune and inflammatory processes relate to a wide spectrum of psychiatric symptoms via central-peripheral interactions informs models that link experiential and environmental factors such as stress to changes in the blood-brain barrier and gut permeability, impacting cognition and mood [74]. The importance of development to these processes is reflected in data showing that depleting the gut microbiota in rodents leads to persistent effects on neuronal function and learning-related plasticity involved in fear-related behaviors and that restoring the microbiota reversed these effects — but only when the restoration was done during the neonatal period [75]. Studies of early life programming of disease risk provide insights into epigenetic mechanisms by which maternal immune activation, stress, and nutrition impact offspring’s long-term metabolic, endocrine, and behavioral outcomes [76]. It has been noted that many of the studies in this rapidly expanding literature do not take into account diagnostic heterogeneity [77]; future work might benefit from the exploration of dimensional approaches and more detailed phenotyping [78].

A second scientific area that is ripe for further research using RDoC-informed approaches is genomics [79]. Evidence of overlap in genomic risk among psychiatric disorders is accumulating. For example, a common variant risk for psychiatric disorders correlates significantly, especially among ADHD, bipolar disorder, and major depressive disorder [80]. Cross-disorder analyses show moderate to high pairwise single nucleotide polymorphism (SNP) based co-heritability between schizophrenia, bipolar disorder, and major depressive disorder [81] and also reveal three clusters of highly genetically related disorders, consisting of mood and psychotic disorders, early-onset neurodevelopmental disorders, and disorders with compulsive behaviors [82]. An analysis of rare de novo coding variants found overlap among obsessive-compulsive disorder, Tourette’s disorder, and autism, suggesting shared biological mechanisms [83].

It is not clear yet whether new approaches to defining neurobehavioral domains or classifying disorders will yield a clearer genomic picture than current diagnostic definitions. It does appear that minimal phenotyping (relying on health records or a small number of self-reported symptoms for case identification) yields less specificity in genetic architecture and lower heritability estimates compared to more detailed phenotyping [84]. An analysis of specific symptoms and clinical features across bipolar disorder and schizophrenia showed over a hundred loci contributing to both disorders, several loci that differentiated between the disorders, and polygenic components that correlated from one disorder to symptoms of the other [85], pointing to the importance of detailed phenotyping for understanding shared versus specific genetic risk. Mobile device-based behavioral testing and clinical assessment using computerized adaptive testing provide opportunities for rapid, low-cost detailed phenotyping appropriate for genomic studies of cross-cutting neurobehavioral domains. McCoy and colleagues [86] used natural language processing to extract five symptom dimensions based on RDoC domains from hospital discharge notes and sought genome-wide association of common variants with these quantitative traits. Loci in three of the five domains were significant, including loci spanning genes associated with neocortical development and neurodegeneration, providing proof-of-principle for this novel approach to identifying dimensional phenotypes for use in psychiatric genetics research.

Finally, in alignment with RDoC’s overarching goal to stimulate research that informs development of more precise and informative classifications and more efficacious and personalized therapeutics, recent findings and novel approaches provide a strong foundation for the next generation of clinical translational work. The shift away from intervention approaches targeting traditional diagnostic categories is reflected in an analysis of trends in mental health clinical trials showing that trials studying non-DSM conditions showed the largest growth of any disorder category from 2007 to 2018 [87]. The fast-fail trial in mood and anxiety spectrum disorders (FAST-MAS) study [4] provides an example of a clinical trial targeting a transdiagnostic neurobehavioral mechanism. Specifically, a kappa-opioid receptor antagonist increased ventral striatum activation during reward anticipation compared to placebo, and this change was associated with improvement in self-reported anhedonia in patients with mood or anxiety disorders. In an example using a different treatment modality, inhibitory trans-cranial magnetic stimulation of the supplementary motor area was shown to ameliorate psychomotor slowing (a behavioral element related to RDoC’s Motor Actions construct) in a sample of patients with schizophrenia or major depressive disorder [88]. The extent to which RDoC has opened the door for new approaches to developing precision treatments is illustrated by a recent commentary regarding preclinical psychopharmacology, recommending that RDoC approaches could be used for preclinical research as part of a number of principles to foster drug discovery [89].

## Conclusions

In the original “Pillars” paper, it was noted that there is no timeframe for the completion of RDoC. Rather, the framework would evolve in response to emerging data and new understandings in order to support a sustained effort toward the ongoing accumulation of knowledge that could bear on the aspirational goal of precision medicine for psychiatry. Achievements in precision therapeutics in other areas of medicine such as oncology and epilepsy [90] provide a roadmap that psychiatry can use as a guide; however, the greatest prior progress has been made in disorders with genetic bases that are directly linked to biological mechanisms, and which allow affordable and precise classification of patients for clinical trials. The small effects of multiple genes for mental disorders provide a daunting contrast for identifying specific pathophysiologies, although slow progress is beginning to emerge [13]. Formidable challenges remain, including those inherent to the etiological, biological, and phenomenological complexity of mental disorders. These have necessitated new conceptual approaches such as RDoC, but also efforts to address more practical hurdles such as the scalability and costliness of deploying clinical neuroscientific tools (e.g., neuroimaging) that impede the integration of translational neuroscience into clinical practice [91].

To address these challenges, three other concurrent NIMH efforts have supported progress toward precision psychiatry. First is the use of experimental therapeutics designs in NIMH-funded clinical trials, which requires a direct test of whether an intervention modifies a pre-specified target and whether doing so affects clinical outcomes; such “target engagement” designs can shed light on pathology-related mechanisms and help match patients to treatments more effectively [4]. Second, the expansion of data sharing for clinical research via the NIMH Data Archive allows combined analyses across common data elements, fostering discovery of novel classifications and clusters. Third, NIMH’s support of research focused on the development and application of computational methods enhances the tools available for the analysis of multivariate and high-dimensional datasets, revealing complex patterns and relationships [85]. In combination with RDoC’s success at shifting the scientific discourse toward dimensional and integrative approaches, these efforts provide a strong foundation for continued progress in understanding and characterizing mental disorders, discovering new causal mechanisms and novel treatment targets, and improving the precision of diagnosis and intervention in the next decade of RDoC.

## Data Availability

Not applicable

## Abbreviations

ADHD: Attention-deficit/hyperactivity disorder
DSM: Diagnostic and Statistical Manual
FAST-MAS: Fast-fail trial in mood and anxiety spectrum disorders
ICD: International Classification of Disease
MDD: Major depressive disorder
NIH: National Institutes of Health
NIMH: National Institute of Mental Health
PRISM: Psychiatric Ratings using Intermediate Stratified Markers
PRS: Polygenic risk score
RDoC: Research Domain Criteria
ROAMER: Roadmap for Mental Health Research in Europe
SNP: Single nucleotide polymorphism
